# Prediction of FAD binding sites in electron transport proteins according to efficient radial basis function networks and significant amino acid pairs

**DOI:** 10.1186/s12859-016-1163-x

**Published:** 2016-07-30

**Authors:** Nguyen-Quoc-Khanh Le, Yu-Yen Ou

**Affiliations:** Department of Computer Science and Engineering, Yuan Ze University, Chung-Li, Taiwan

**Keywords:** Electron transport protein, FAD binding site, Transporter, Annotation, Feature selection, Position specific scoring matrix, Significant amino acid pairs

## Abstract

**Background:**

Cellular respiration is a catabolic pathway for producing adenosine triphosphate (ATP) and is the most efficient process through which cells harvest energy from consumed food. When cells undergo cellular respiration, they require a pathway to keep and transfer electrons (i.e., the electron transport chain). Due to oxidation-reduction reactions, the electron transport chain produces a transmembrane proton electrochemical gradient. In case protons flow back through this membrane, this mechanical energy is converted into chemical energy by ATP synthase. The convert process is involved in producing ATP which provides energy in a lot of cellular processes. In the electron transport chain process, flavin adenine dinucleotide (FAD) is one of the most vital molecules for carrying and transferring electrons. Therefore, predicting FAD binding sites in the electron transport chain is vital for helping biologists understand the electron transport chain process and energy production in cells.

**Results:**

We used an independent data set to evaluate the performance of the proposed method, which had an accuracy of 69.84 %. We compared the performance of the proposed method in analyzing two newly discovered electron transport protein sequences with that of the general FAD binding predictor presented by Mishra and Raghava and determined that the accuracy of the proposed method improved by 9–45 % and its Matthew’s correlation coefficient was 0.14–0.5. Furthermore, the proposed method enabled reducing the number of false positives significantly and can provide useful information for biologists.

**Conclusions:**

We developed a method that is based on PSSM profiles and SAAPs for identifying FAD binding sites in newly discovered electron transport protein sequences. This approach achieved a significant improvement after we added SAAPs to PSSM features to analyze FAD binding proteins in the electron transport chain. The proposed method can serve as an effective tool for predicting FAD binding sites in electron transport proteins and can help biologists understand the functions of the electron transport chain, particularly those of FAD binding sites. We also developed a web server which identifies FAD binding sites in electron transporters available for academics.

## Background

**Fig. 1:**
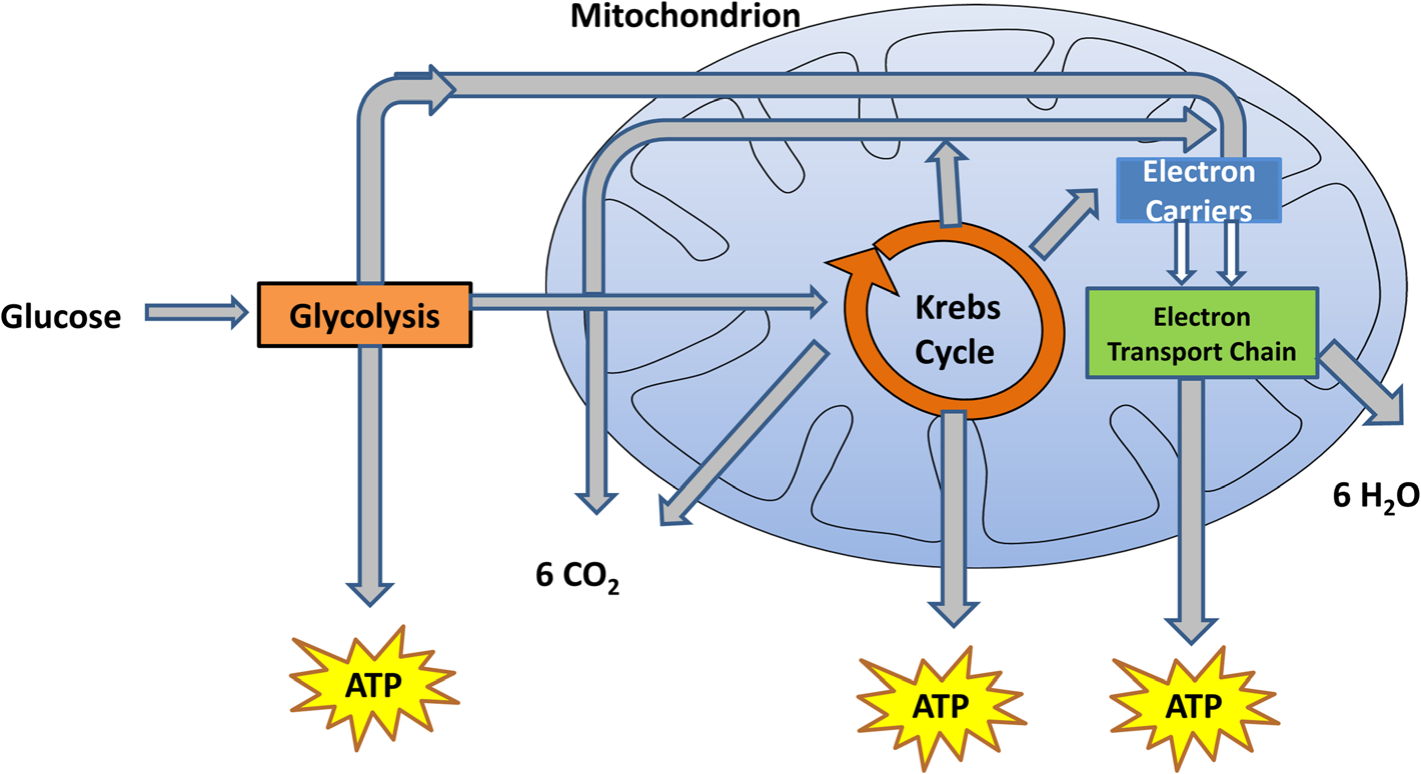
Cellular respiration process

Cellular respiration is the process for producing adenosine triphosphate (ATP) and enables cells to obtain energy from foods. During cellular respiration, cells break down food molecules, such as sugar, and release energy. The objective of cellular respiration is to harvest electrons from organic compounds to create ATP, which is used to provide energy for most cellular reactions. Figure 1 shows the architecture of the cellular respiration process.

As cells undergo cellular respiration, they require a pathway to store and transport electrons (i.e., the electron transport chain). The electron transport chain components are organized into four complexes (Complex I, Complex II, Complex III, and Complex IV) and ATP synthase (which can be called Complex V). The process of electron transport chain starts from the mitochondrial inner membrane, which electrons transfer from Complex I with nicotinamide adenine dinucleotide (NADH) and succinate (Complex II) to oxygen. In the next step, a carrier (coenzyme Q) that embeds in the cell membrane receives electrons from complex I and pass to Complex III (cytochrome b, c1 complex). Electrons bypass Complex II, the succinate dehydrogenase complex, which is an independent starting stage and is not a component of the NADH pathway. The pathway from Complex III leads to cytochrome c then moves to Complex IV (cytochrome oxidase complex). In the final step, ATP synthase is active by the proton electrochemical to utilize the flow of H+ to generate ATP, which provides energy in numerous cellular processes.

Flavin adenine dinucleotide is one of the most vital molecules in the electron transport chain. It is mainly in Complex II, which is an enzyme complex bound to the inner mitochondrial membrane of mammalian mitochondria and many bacterial cells. Regarding the reaction mechanism of Complex II, succinate is bound and a hydride is transferred to FAD to generate FADH2. After the electrons are derived from succinate oxidation through FAD, they tunnel along the [Fe-S] relay to the [3Fe-4S] cluster. These electrons are subsequently transferred to an awaiting ubiquinone molecule within the active site. The fundamental role of Complex II in the electron transfer chain of mitochondria renders it vital in most organisms, and removing Complex II from the genome has been shown to be lethal at the embryonic stage in mice.

Predicting FAD binding sites in electron transporters is vital for helping biologists clearly understand the operating mechanisms of the electron transport chain and Complex II. In this study, we developed a method that is based on position specific scoring matrix (PSSM) profiles and significant amino acid pairs (SAAPs) for identifying FAD binding residues in electron transport proteins.

FAD binding sites have attracted the interest of numerous researchers because of their relevance in electron transport chains. Prominent studies conducted on FAD binding sites include those by Mishra and Raghava [1] and Fang [2]. Mishra and Raghava [1] used support vector machines to predict FAD binding residues. They also developed a free web server for identifying FAD binding residues in specific sequences. Moreover, Fang [2] used evolutionary information to improve the prediction performance.

Numerous studies have also been conducted on transport proteins. For example, Saier [3] provided a web database containing the sequence, classification, structural, and evolutionary information of transport systems from various living organisms. Furthermore, Ren [4] presented transportDB, which is a comprehensive database of transporters and outer membrane channels. Chen [5] divided electron transport targets into four types of transport proteins to conduct prediction and analysis. After the prediction and analysis, Chen classified the transport proteins and determined the functions of each protein type in the transport protein. Ou [6] attempted to discriminate metal-binding sites in electron transport by using radial basis function networks (RBFNs).

The current study proposes an approach based on PSSM profiles and SAAPs for identifying FAD binding sites in electron transport proteins. We used a set of 55 FAD binding proteins as the training data set and six FAD binding proteins in electron transport proteins as an independent data set. We applied the independent data set to evaluate the performance of the proposed method, which demonstrated an accuracy of 69.84 %. Compared with the general FAD binding predictor developed by Mishra and Raghava, the proposed method exhibited a 9 %–45 % improvement in accuracy and Matthew’s correlation coefficient (MCC) of 0.14–0.5 when applied to two newly discovered electron transport protein sequences. The proposed method also reduces the number of false positives significantly and offers useful information for biologists. The proposed method can serve as an effective tool for predicting FAD binding sites in electron transport proteins and can help biologists understand electron transport chain functions, particularly those of FAD binding sites.

## Methods

**Fig. 2:**
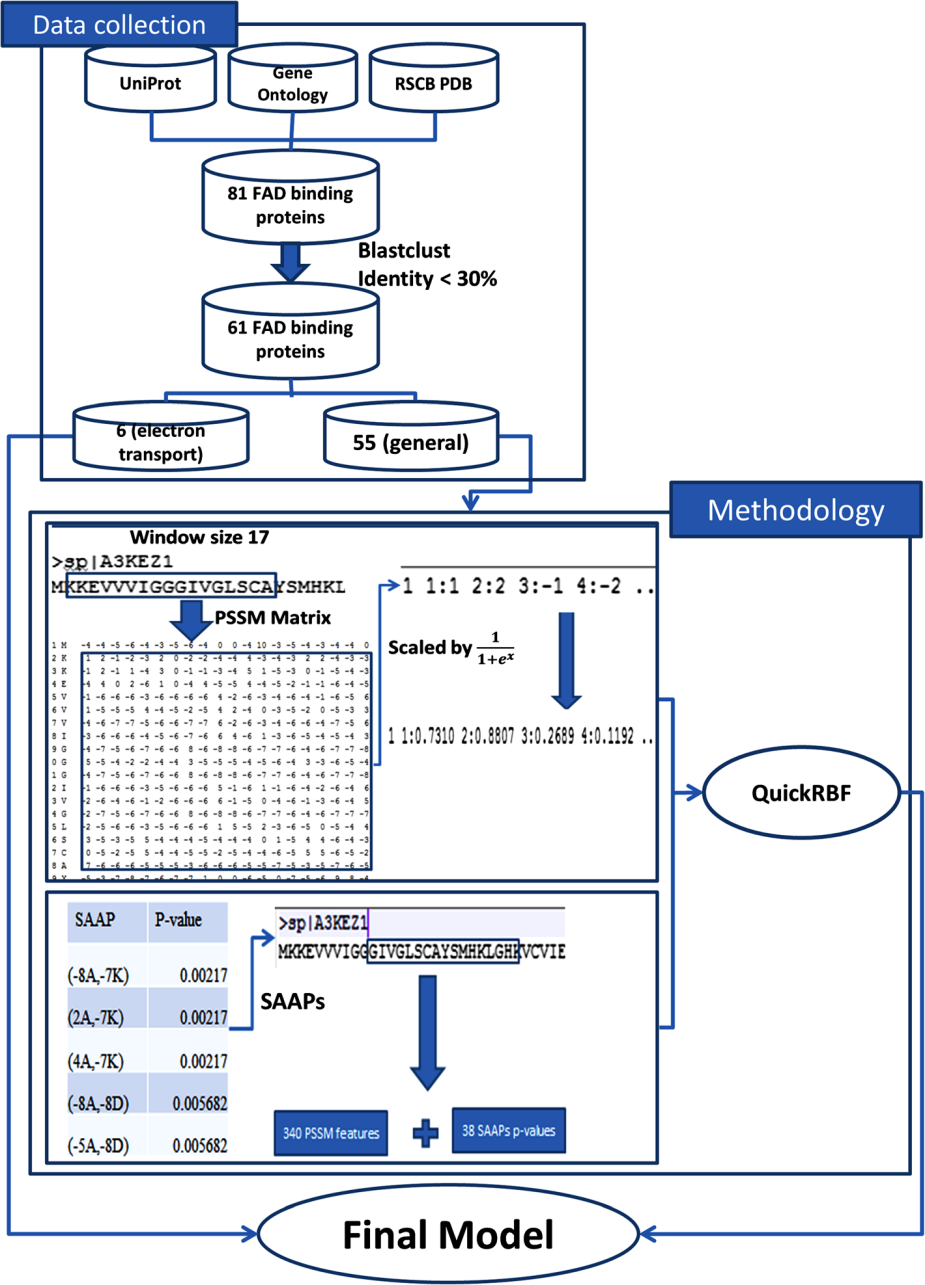
Flowchart of the proposed method for identifying FAD binding sites in electron transport proteins

This study focused on identifying FAD binding sites in electron transport proteins. Figure 2 illustrates a flowchart of the study, which included three subprocesses in each phase: data collection, feature set generation, and model evaluation. According to this flowchart, we developed a novel approach that is based on PSSM profiles and SAAPs for predicting FAD binding sites in electron transport proteins. The details of the proposed approach are described as follows.

### Data set

First, we collected data about transport proteins and electron transport proteins from the UniProt [7] database. Subsequently, we removed sequences without the annotation “evidence at protein level” or “complete.” After this exclusion, 6694 transport proteins and 889 electron transport proteins remained and were surveyed. Next, we retrieved all FAD binding sites in the electron transport proteins. We collected data on only nine FAD binding proteins. However, creating a precise model requires using a higher number of proteins; thus, we collected data on additional general FAD binding proteins from other sources. We retrieved data from the Gene Ontology (GO) [8] and Protein Data Bank (PDB) [9, 10] databases by using the molecular function of FAD binding. In the GO database, we applied three molecular functions of FAD binding: GO:0050660 (FAD binding), GO:0071949 (FAD binding), and GO:0071950 (FADH_2_ binding). From these three molecular functions, we obtained data on a total of 42 FAD binding proteins. We applied the same approach to the PDB database and obtained data on a total of 72 FAD binding proteins. We removed duplicated proteins and 81 general FAD binding proteins remained. Next, BLAST [11] was applied to exclude sequences with a sequence identity of more than 40 % from the data set. Finally, 61 FAD binding proteins were used in this study (Table 1).

**Table 1:** Statistics of all retrieved FAD binding proteins with FAD and non-FAD binding sites

	Number of proteins	FAD binding sites	Non-FAD binding sites
FAD binding in electron transport	6	63	3030
General FAD binding proteins	55	940	26475

We divided the collected protein sequences into two data sets: training and independent test data sets. In this phase, the training data set was used for identifying FAD binding sites, and the independent test data set was used for evaluating the performance of the proposed method. We used all six FAD binding proteins in the electron transport chain as the independent data set; thus, the training data set comprised 55 general FAD proteins (containing 863 FAD binding sites and 24408 non-FAD binding sites). Table 2 lists the details of all data sets.

**Table 2:** Details of all 61 FAD binding proteins with a UniProt ID in the present study (six FAD binding proteins in electron transport served as an independent data set)

Independent dataset	Training dataset				
P00455	O95831	P21890	P08165	Q5SJP8	Q92947
Q03103	P00371	P26440	Q5SH33	Q5SK63	Q945K2
Q96HE7	P00390	P37747	P66004	Q5UVJ4	Q96329
Q9YHT1	P07342	P38038	Q0QLF4	Q709F0	Q9AL95
P55931	O53355	P39662	Q28943	Q7SID9	C6ELC9
A3KEZ1	O54050	P41367	P97275	Q7WZ62	D0VWY5
	O60341	P45954	Q2GBV9	Q7X2H8	O52582
	P0A6U3	P47989	Q389T8	Q7ZA32	Q9RSY7
	P15651	P49748	Q47PU3	Q8DMN3	Q9UBK8
	P19920	P55789	Q52437	Q8X1D8	Q9UKU7
	P07872	P09622	Q9HJI4	Q9HKS9	Q9HTK9

### Sequence information

Sequence information is one of the first features set in predicting the secondary structure of proteins [12, 13]. In this feature, each amino acid sequence is represented by a number 0 or 1, creating a binary matrix. From the binary matrix, the value for each amino acid can be calculated. For example, if the sequence of amino acids is ARNDCQEGHILKMFPSWYV and the value for amino acid N must be calculated, the third position is set to 1 and the others are set to 0. In this study, we also used two types of advance sequence information, namely PAM250 and BLOSUM62.

### PAM250

A percent accepted mutation (PAM) [14] matrix represents the elements involved in the conversion of amino acids into amino acids within a variable probability of evolutionary distance. A PAM matrix was created in the protein sequence alignment and various phylogenetic trees with the assumption that amino acids are amino acids and that each amino acid is substituted with another amino acid, to establish an acceptable point mutation matrix (accepted point mutation matrix).

A matrix is usually employed to mark aligned peptide sequences in order to identify the similarity of such sequences. By comparing aligned protein sequences with a known homology and determining the “accepted point mutations”, the aforementioned numbers were derived. The frequencies of such mutations were arranged in a table as a “log odds matrix”:

$$ {\mathrm{M}}_{\mathrm{ij}} = 10\left( \log 10{\mathrm{R}}_{\mathrm{ij}}\right), $$

where M_ij_ is the matrix component and R_ij_ is the probability of that substitution, then divided by the standardized frequency of amino acid sequences. Note that all the numbers are rounded to the integer number. The base-10 log is utilized so that the numbers can be added instead of multiplied to decide the score of a practical set of sequences.

### BLOSUM62

The block substitution matrix (BLOSUM) [15] is used to assess differences in effectiveness between evolutions of protein sequence alignment methods. They are retrieved from the BLOCKS database, and some of the protein amino acid sequences are retained; the calculated relative amino acid is replaced by the calculated frequency and probability. A BLOSUM62 matrix is commonly collected in a database sequence BLOCKS with 62 % sequence similarity, and the sequence is then deduced from a score matrix.

### PSSM profiles

PSSM is a matrix commonly used for representing motifs in biological sequences [16]. It is a matrix of score values and provides a weighted match to any specific substring of fixed length. This matrix has one row for each letter of the alphabet and one column for each position in the pattern.

In recent years, the PSSM has widely been considered an indicator of the properties of protein sequences. The PSSM is used in determining the evolution of sequence information in a specific location as well as the amino acid replacement ratio to identify protein sequences; such sequences represent the original 20 amino acid types in the protein and are used to replace an amino acid with its degree of influence. The PSSM has been extensively used for predicting the secondary structure of proteins as well as subcellular locations and other biological information, and it has been reported to produce favorable results.

We collected all sequence data from BLAST [11] and the non-redundant protein database and used them to establish the sequences in a PSSM. After the PSSM sequences were established, we calculated the optimal protein sequence for each amino acid. We placed 20 types of amino acids in the calculated sequences, leading to the creation of a matrix. If a window size of 17 is used, then the matrix size is 17 * 20 = 340 (because the calculated value for each amino acid was 20). This matrix should be added to predict the properties of the protein sequence. Identical amino acid residues can be replaced with a specific value of amino acids. We used the following numerical normalization formula to convert the values to values between 0 and 1:

$$ \mathrm{F}\kern0.5em \left(\mathrm{x}\right)=\frac{1}{1+ \exp \left(\mathit{\hbox{-}}x\right)} $$

### F-score

In binary classification analysis, an F-score is a simple parameter applied for measuring the accuracy of a test by using two sets of real numbers [17]. The F-score is defined as follows:

$$ F(i)=\frac{{\left({\overline{x}}_i^{\left(+\right)}-{\overline{x}}_i\right)}^2+{\left({\overline{x}}_i^{\left(-\right)}-{\overline{x}}_i\right)}^2}{\frac{1}{n_{+}-1}{\displaystyle {\sum}_{k=1}^{n+}{\left({x}_{k,i}^{\left(+\right)}-{\overline{x}}_i^{\left(+\right)}\right)}^2}+\frac{1}{n_{-}-1}{\displaystyle {\sum}_{k=1}^{n-}{\left({x}_{k,i}^{\left(-\right)}-{\overline{x}}_i^{\left(+\right)}\right)}^2}} $$

where n^+^ is the number of positive instances and n^−^ is the number of negative instances. Furthermore, ${\overline{x}}_{\mathrm{i}},\kern0.5em {\overline{x}}_i^{\left(+\right)}$, and ${\overline{x}}_i^{\left(-\right)}$ are the averages of the *i*th feature of the entire, positive, and negative data sets, respectively; x^(+)^_k,i_ is the *i*th feature of the *k*th positive instance; and x^(−)^_k,i_ is the *i*th feature of the *k*th negative instance. We calculated all F-score values for all feature sets of FAD binding sites in electron transport proteins. A higher F-score indicates that the corresponding feature has a higher amount of special information. Therefore, we added the F-score values to the PSSM features. In this study, we added the 30 highest F-scores to the PSSM features.

### Significant amino acid pairs

**Fig. 3:**
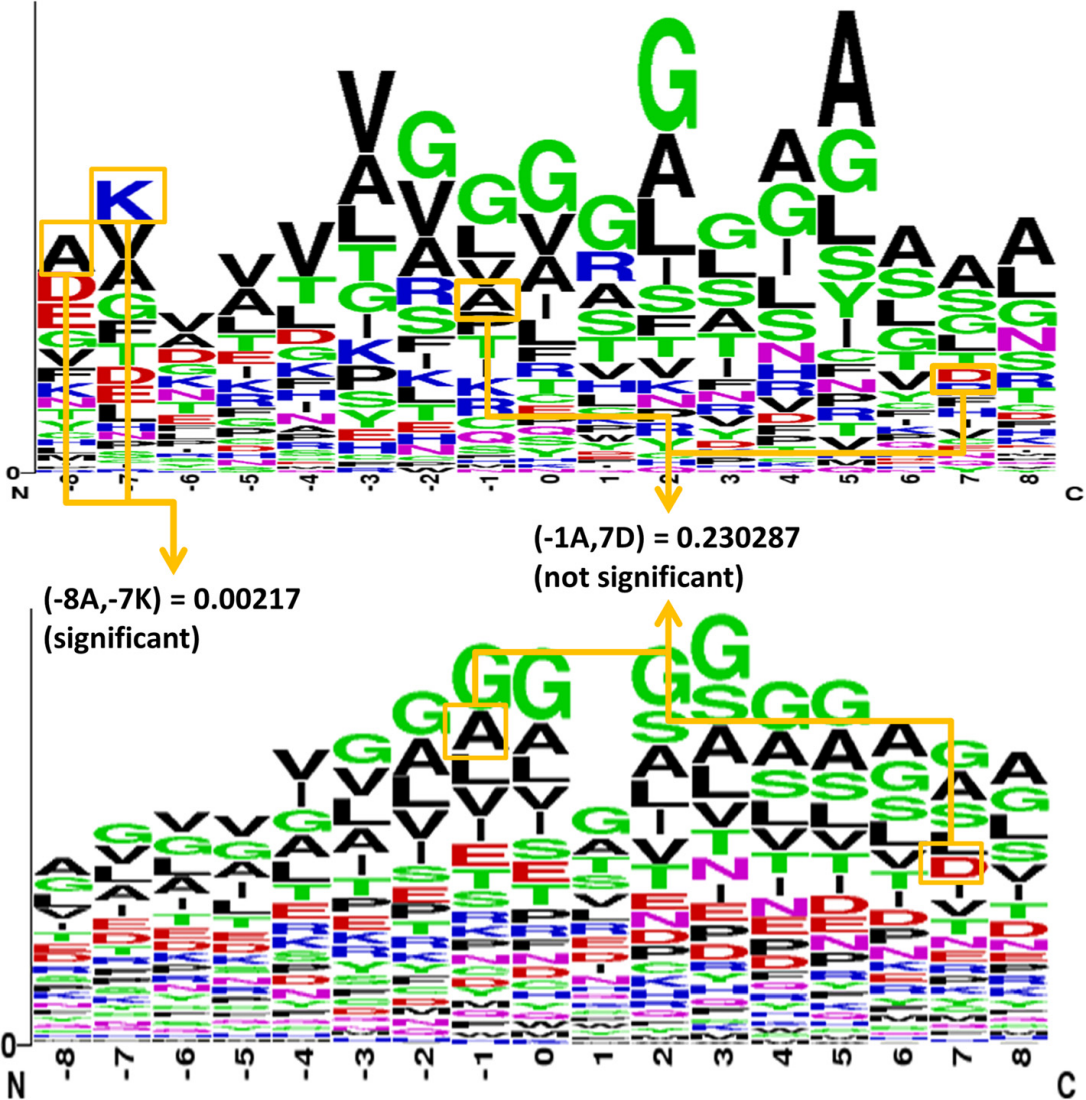
Proposed method for calculating initial SAAP values

We adopted SAAPs to improve the performance of the proposed method in predicting FAD binding sites in electron transport proteins. The SAAPs around the FAD binding sites were identified on the basis of six FAD binding proteins, and the remaining SAAPs were identified on the basis of a statistical distribution measurement. Each amino acid pair surrounding FAD binding sites was calculated using a *p*-value:

$$ \mathrm{p}\hbox{-} {\mathrm{value}}_{\mathrm{k}}=\frac{\left(\begin{array}{c}\hfill \mathrm{M}\hfill \\ {}\hfill \mathrm{x}\hfill \end{array}\right)\left(\begin{array}{c}\hfill \mathrm{N}\hbox{-} \mathrm{M}\hfill \\ {}\hfill \mathrm{n}\hbox{-} \mathrm{x}\hfill \end{array}\right)}{\left(\begin{array}{c}\hfill \mathrm{N}\hfill \\ {}\hfill \mathrm{n}\hfill \end{array}\right)}, $$

where N denotes the number of sequences in the entire data set, M denotes the number of sequences in the positive data set, and (N-M) denotes the number of sequences in the negative data set; n, x, and n-x denote the number of sequences including a *k*th SAAP in the entire data set, positive data set, and negative data set. Figure 3 shows the method used for calculating the *p*-value from FAD binding sites in electron transport chains.

A *p*-value less than 0.13 indicates that the amino acid pair surrounding FAD binding sites is significant. That is, numerous special features exist, with some features having a *p*-value less than 0.13. After we calculated the *p*-values for all amino acid pairs surrounding FAD binding sites with a window size of 17, we added the ranked SAAPs to the feature set in descending order. Finally, 38 SAAPs were added to the feature set of FAD binding sites in electron transport proteins.

### Radial basis function networks

**Fig. 4:**
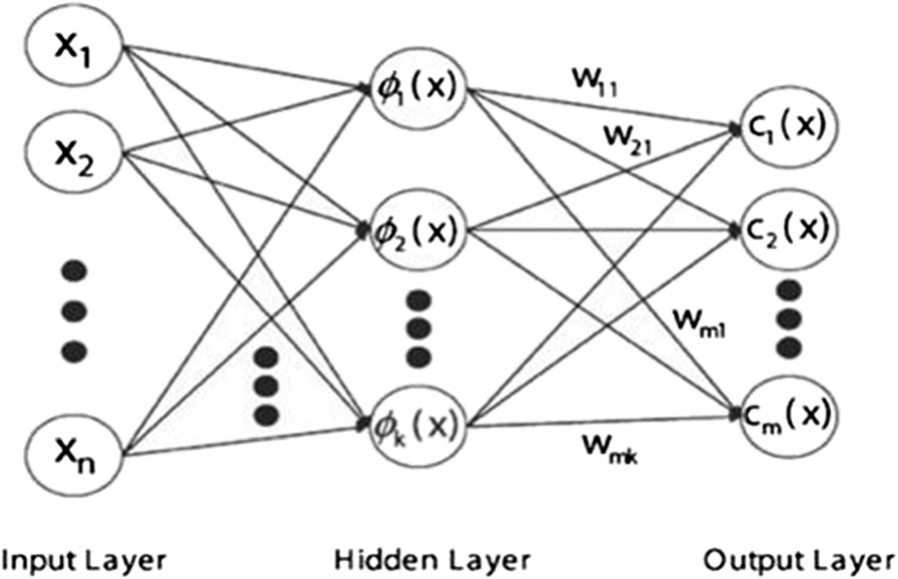
Architecture of the RBFN

We employed the QuickRBF package [18] to construct RBFN classifiers. Figure 4 shows the architecture of the RBF network. Furthermore, we assigned a constant bandwidth of 5 for each kernel function in the network. We also used all training data as centers. Subsequently, the RBFN classifier was used to identify FAD binding sites according to the output function value. We explained the details of the network structure and design in our previous article [19].

RBFN-based classifications have been used in several applications in bioinformatics to predict cleavage sites in proteins [20], interresidue contacts [21], and protein disorder [22]; furthermore, they have been applied for discriminating β-barrel proteins [23], classifying transporters [24, 25], identifying O-linked glycosylation sites [26], and identifying ubiquitin conjugation sites [27].

The general mathematical form of output nodes in an RBFN is expressed as follows:

$$ {g}_j(x)={\displaystyle \sum_{i=1}^k{w}_{ji}\varphi \left(\left\Vert x-{\mu}_i\right\Vert; {\sigma}_i\right);} $$

where *g*_*j*_(*x*) is the function corresponding to the *j*th output node and is a linear combination of *k* radial basis functions $\varphi \left(\right)$ with center m_*i*_ and bandwidth s_*i*_; in addition, w_*ji*_ is the weight associated with the correlation between the jth output node.

### Assessment of predictive ability

We measured the predictive performance of the proposed method by using sensitivity, specificity, accuracy, and MCC metrics. TP, FP, TN, and FN represent true positive, false positive, true negative, and false negative, respectively.

#### Sensitivity

This parameter enables computing the percentage of accurately predicted FAD binding sites.

$$ \mathrm{Sensitivity}=\frac{\mathrm{TP}}{\mathrm{TP}+\mathrm{F}\mathrm{N}} $$

#### Specificity

This parameter enables computing the percentage of accurately predicted non-FAD binding sites.

$$ \mathrm{Specificity}=\frac{\mathrm{TN}}{\mathrm{TN}+\mathrm{F}\mathrm{P}} $$

#### Accuracy

This parameter enables computing the percentage of accurately predicted FAD and non-FAD binding sites.

$$ \mathrm{Accuracy}=\frac{\mathrm{TP}+\mathrm{T}\mathrm{N}}{\mathrm{TP}+\mathrm{F}\mathrm{P}+\mathrm{T}\mathrm{N}+\mathrm{F}\mathrm{N}} $$

#### MCC

This parameter represents the quality of prediction and is used for resolving imbalance in data sets. An MCC value of 1 indicates a perfect prediction.

$$ \mathrm{M}\mathrm{C}\mathrm{C}=\frac{\mathrm{TP}\times \mathrm{T}\mathrm{N}\hbox{-} \mathrm{F}\mathrm{P}\times \mathrm{F}\mathrm{N}}{\sqrt{\left(\mathrm{T}\mathrm{P}+\mathrm{F}\mathrm{P}\right)\left(\mathrm{T}\mathrm{P}+\mathrm{F}\mathrm{N}\right)\left(\mathrm{T}\mathrm{N}+\mathrm{F}\mathrm{P}\right)\left(\mathrm{T}\mathrm{N}+\mathrm{F}\mathrm{N}\right)}} $$

## Results and discussion

### Amino acid composition analysis

**Fig. 5:**
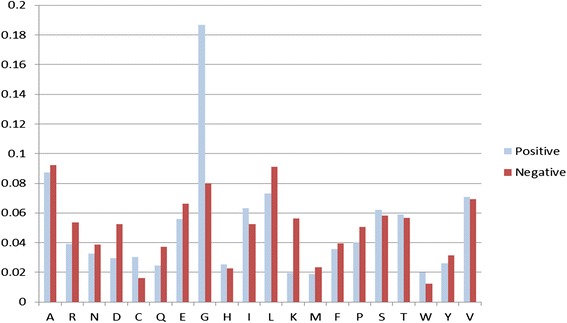
Amino acid composition of FAD binding interacting residues and noninteracting residues in 55 general FAD binding proteins

**Fig. 6:**
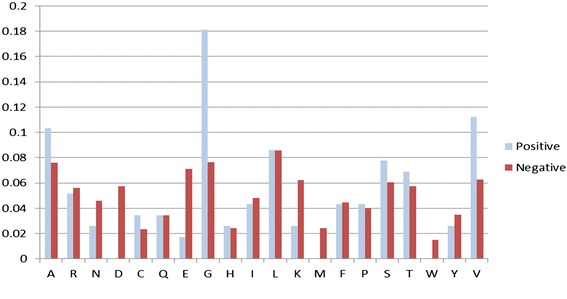
Amino acid composition of FAD interacting residues and noninteracting residues in six FAD binding proteins in the electron transport chain

We analyzed the composition of interacting and non-interacting FAD binding sites by computing the occurrence frequency of amino acids in these sites. Regarding the interacting FAD binding sites, the amino acids G, S, A, and T exhibited the significantly highest occurrence frequency in two interaction instances (general FAD binding proteins and FAD binding proteins in electron transport proteins) (Figs. 5 and 6). We inferred that glycine is vital for the interaction with FAD binding sites. Regarding non-interacting binding sites, the amino acids A, L, and G exhibited the highest occurrence frequency in both instances.

**Fig. 7:**
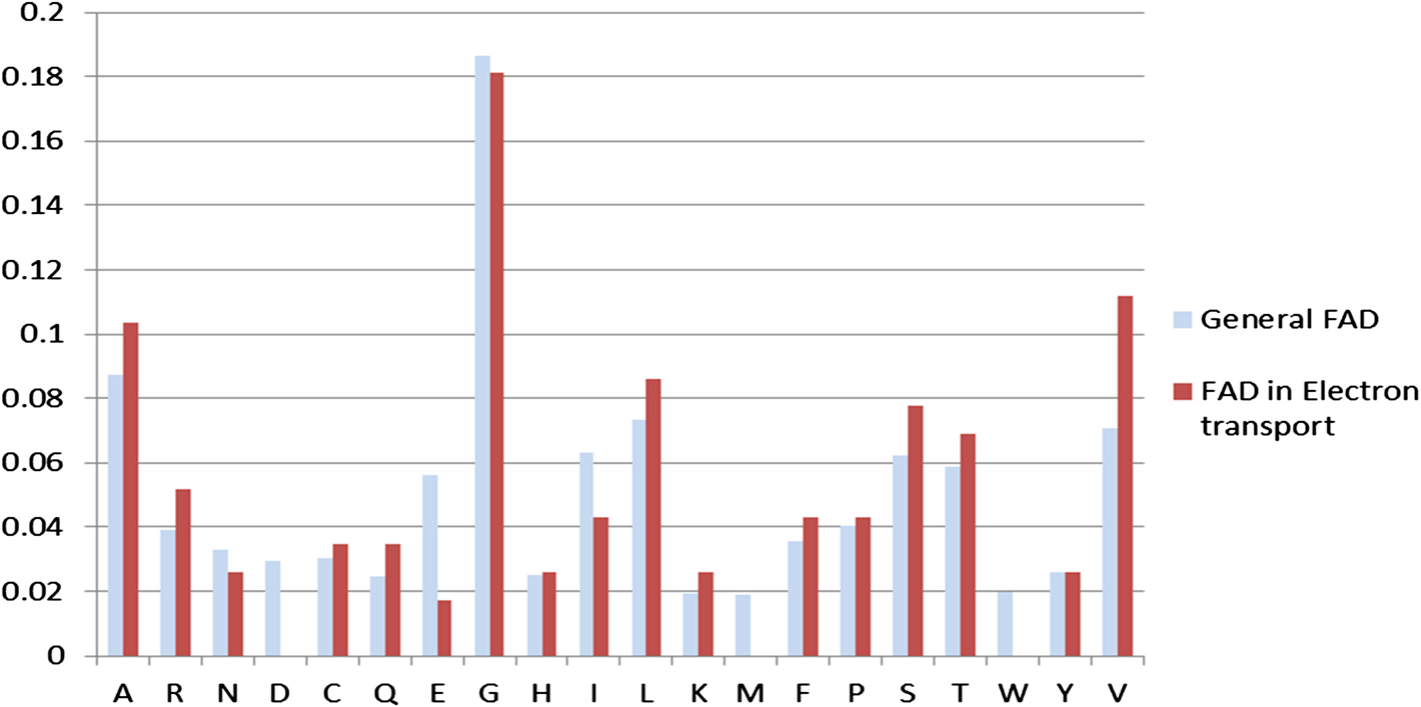
Comparison of percentage composition of FAD interacting residues in six FAD binding proteins in the electron transport chain and 55 general FAD binding proteins

Figure 7 shows a comparison between general FAD binding proteins and FAD binding proteins in electron transport proteins. We observed some differences between the two types of proteins, and the amino acids V, E, and I exhibited considerable differences.

### Performance in predicting FAD binding sites in electron transport proteins by using various window sizes

We created an FAD binding classifier by using the 61 FAD binding proteins collected. We applied the QuickRBF classifier by using window sizes ranging from 13 to 19 for comparison (Table 3). We measured the predictive performance of the proposed PSSM-based method. As shown in Table 3, changing the window size did not exert considerable effects on the result. The result obtained when the window size was set to 17 was favorable, and the measured sensitivity, specificity, accuracy, and MCC were approximately 80.8 %, 80.2 %, 80.2 %, and 0.27, respectively. Although the MCC was low, all the other performance metrics were approximately 80. We used the experiment with a window size of 17 to create the FAD binding classifier model.

**Table 3:** Comparison of performance in identifying FAD binding sites in the electron transport chain with different window sizes

	Window Size	True positive	False positive	True negative	False negative	Sens	Spec	Acc	MCC
5-fold	WS13	139	973	3909	33	80.8	80.1	80.1	0.27
	WS15	139	990	3893	33	80.8	79.7	79.8	0.26
	WS17	139	966	3917	33	80.8	80.2	80.2	0.27
	WS19	138	1004	3879	35	79.8	79.4	79.5	0.26
indept	WS13	50	1444	1586	13	79.4	52.3	52.9	0.09
	WS15	50	1223	1807	13	79.4	59.6	60	0.11
	WS17	51	1169	1861	12	81	61.4	61.8	0.12
	WS19	51	1225	1805	12	81	59.6	60	0.12

**Fig. 8:**
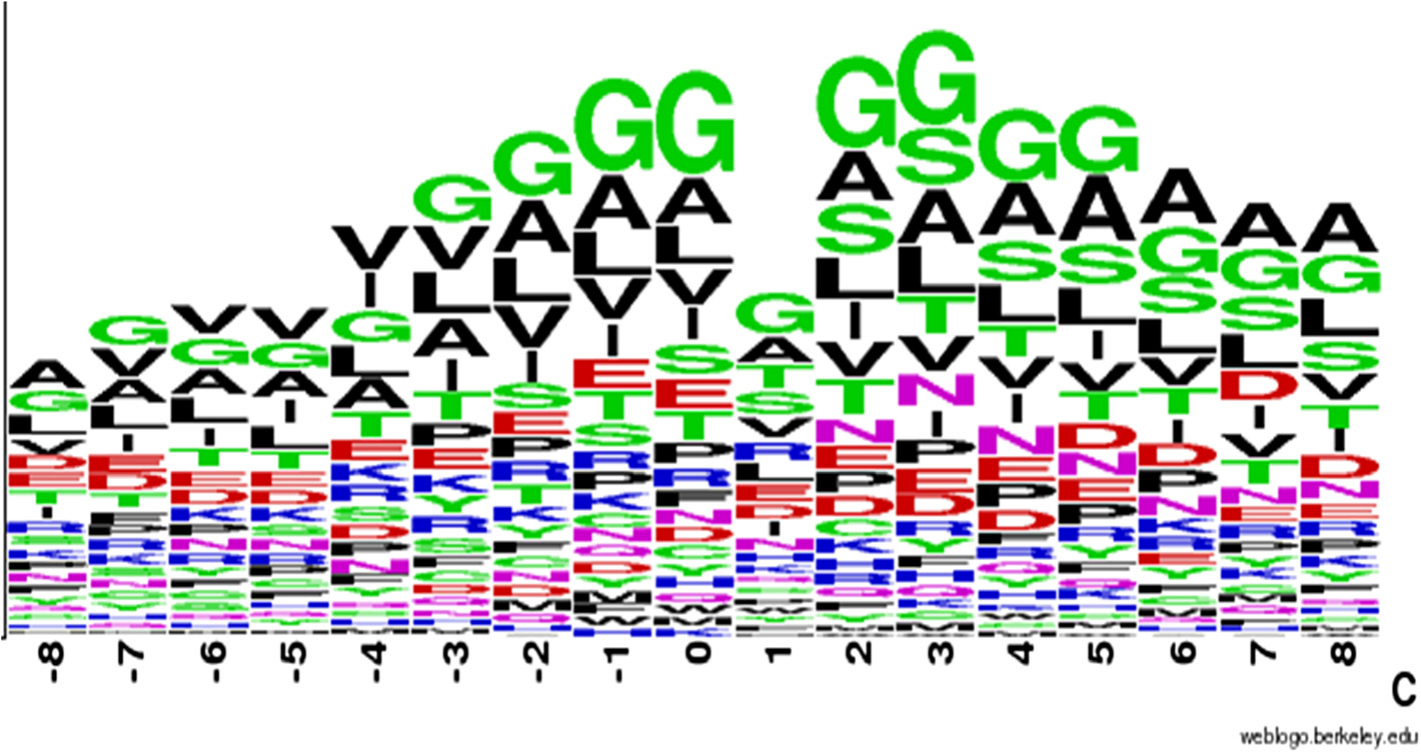
Sequence logo for 55 general FAD binding proteins (generated from WebLogo)

**Fig. 9:**
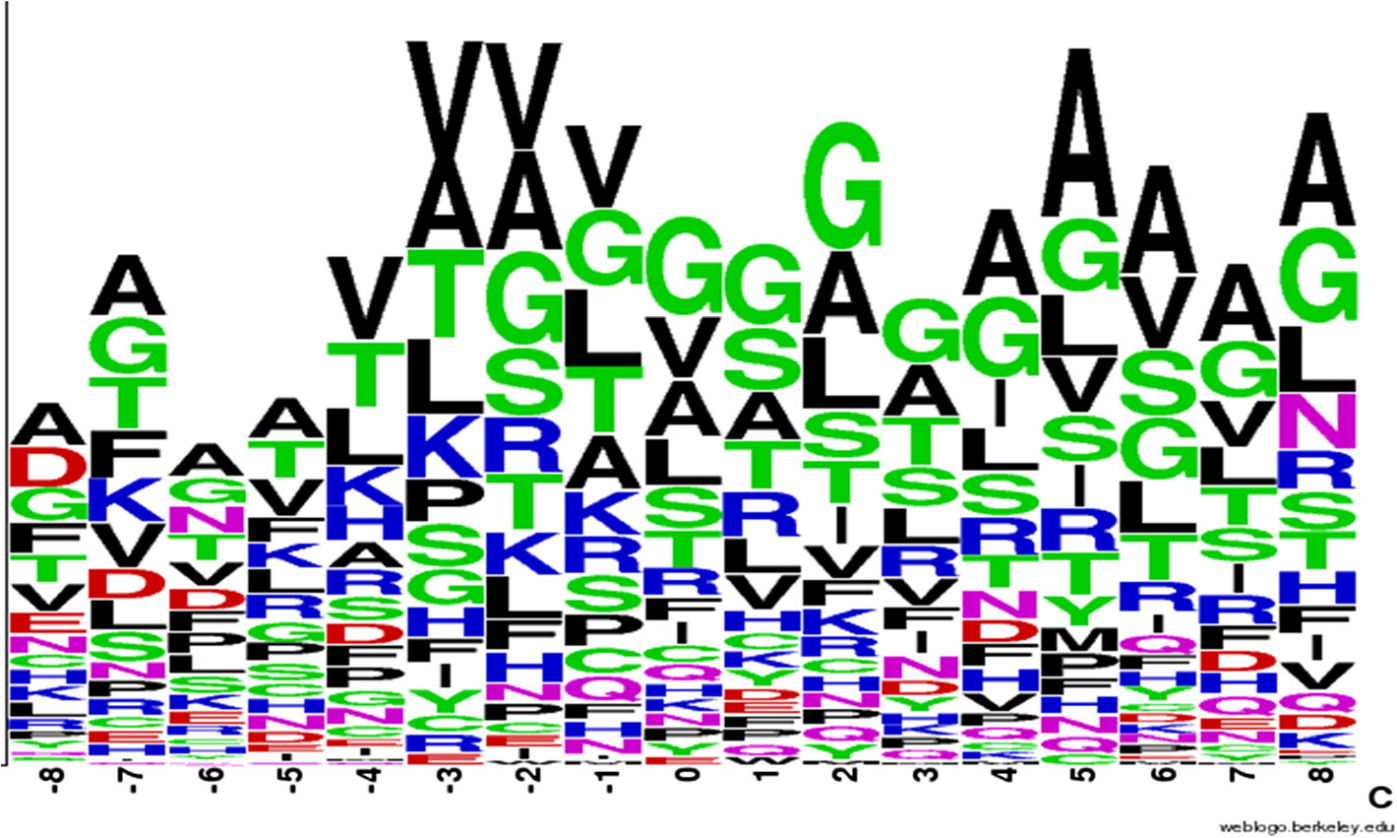
Sequence logo for six FAD binding proteins in the electron transport chain (generated from WebLogo)

As shown in Figs. 8 and 9, the sequence frequency logo was generated using a tool provided by the WebLogo server [28]. The window size was set to 17 and used to confirm the FAD binding fragment for comparison. These two figures indicate that some differences exist between the general FAD binding proteins and FAD binding proteins in the electron transport chain. For example, the amino acids T, K, I, and R exhibited clear differences at positions ranging from −4 to −1.

### Performance in predicting FAD binding sites in electron transport proteins with different feature sets

Table 4 shows the performance assessment results obtained by discriminating FAD binding sites in electron transport chains with different feature sets. We used the established FAD classifier to predict our independent data set (six FAD binding proteins in the electron transport chain) by setting the window size to 17. As shown in Table 4, the predictive performance of the proposed method was more favorable than that of the other methods (i.e., BINARY, BLOSUM62, PAM250, and F-Score). Although the performance of the proposed method was not extremely high (sensitivity = 80.95 %, specificity = 69.6 %, accuracy = 69.84 %, and MCC = 0.15), it was still superior to that of the other methods. We observed that the performance improved when we added SAAPs from FAD binding proteins in the electron transport chain to the PSSM. Thus, the proposed method was effective in predicting FAD binding sites in electron transport proteins.

**Table 4:** Comparison of performance in identifying FAD binding sites in the electron transport chain with different feature sets

Feature set	True positive	False positive	True negative	False negative	Sens	Spec	Acc	MCC
BINARY	45	972	2058	18	71.43	67.92	67.99	0.12
BLOSUM62	41	977	2053	22	65.08	67.76	67.7	0.1
PAM250	42	996	2032	21	66.67	67.11	67.1	0.1
PSSM	51	1169	1861	12	80.95	61.42	61.82	0.12
PSSM + F-score	51	1142	1888	12	80.95	62.31	62.69	0.13
PSSM + SAAPs	54	1074	1955	9	85.71	64.54	64.97	0.15

### Significance analysis based on the proposed method

**Fig. 10:**
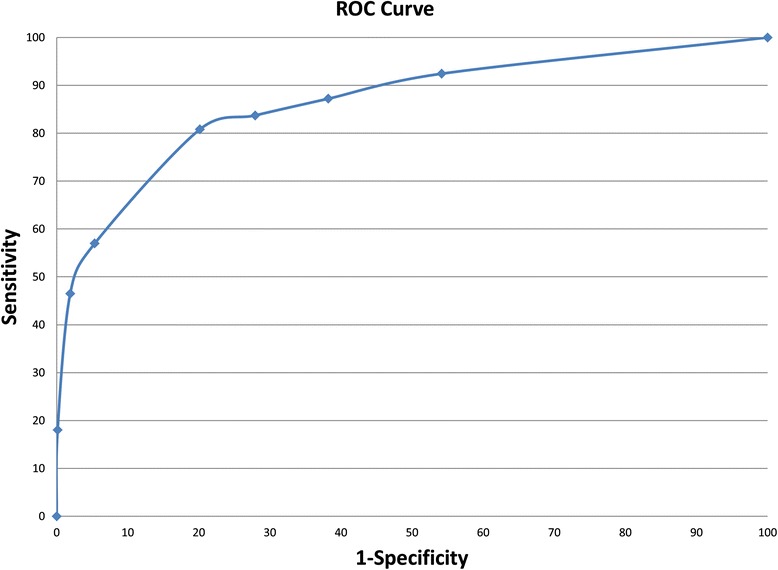
ROC Curve for performance of predicting FAD binding sites in electron transport proteins with PSSM and SAAPs

Receiver operating characteristic (ROC curve) and area under the curve (AUC) are also used in presenting the accuracy of the test in the presented results [29, 30]. Figure 10 plots the ROC curve based on Sensitivity and Specificity of our prediction results. According to the ROC curve, we calculated the AUC to measure the accuracy. The AUC from this study reached 0.8618325, and therefore we can use this model to identify FAD binding sites in the electron transport chains with good results.

### Performance in predicting FAD binding sites in electron transport proteins with different classifiers

Table 5 shows the performance assessment results obtained by discriminating FAD binding sites in electron transport chains with different classifiers. We applied our method in independent dataset with different classifiers, i.e., kNN, RandomForest (in WEKA package [31, 32]) and LibSVM classifiers [33]. The results show in Table 5 can prove that our classifier perform well than the others. Therefore we can use our method to identify FAD binding sites in electron transport proteins with high results.

**Table 5:** Comparison of performance in identifying FAD binding sites in the electron transport chain with different classifiers

Classifier	True positive	False positive	True negative	False negative	Sens	Spec	Acc	MCC
kNN	45	1448	1581	9	85.71	52.2	52.88	0.11
RandomForest	31	1055	1974	32	49.21	65.17	64.84	0.04
LibSVM	53	1149	1880	10	84.13	62.07	62.52	0.13
QuickRBF	54	1074	1955	9	85.71	64.54	64.97	0.15

### Leave-one-out analysis with six FAD binding proteins in electron transport chains

Table 6 shows the final results obtained from a leave-one-out analysis of six FAD binding proteins in electron transport chains. Although the number of proteins used in the experiment was not high, we conducted this experiment to obtain a reference for comparison and validate the performance of the proposed method in predicting FAD binding sites in electron transport chains. The analysis results revealed that the proposed method performed well, exhibiting an average sensitivity of 97.37 %, average specificity of 96.36 %, average sensitivity of 96.39 %, and average MCC of 0.66.

**Table 6:** Comparison of performance in identifying FAD binding sites in the electron transport chain with PSSM and SAAPs

Protein	True positive	False positive	True negative	False negative	Sens	Spec	Acc	MCC
Q9YHT1	17	223	417	9	65.38	65.16	65.17	0.12
P00455	9	111	247	3	75	68.99	69.19	0.17
Q03103	6	1	557	0	100	99.82	99.82	0.92
Q96HE7	6	68	394	1	85.71	85.28	85.29	0.24
A3KEZ1	6	5	400	0	100	98.77	98.78	0.73
P55931	6	0	612	0	100	100	100	1

### Comparison of the proposed method with another method

We compared the performance of the proposed method with that of the FADPred approach presented by Mishra and Raghava [1]. In this comparison, in addition to the six FAD binding proteins in the electron transport chain, we used two new proteins, namely Q96HE7 and A3KEZ1, which have been demonstrated in experiments conducted after 2010. We subsequently evaluated the results of the proposed method in analyzing these two proteins and compared them with results of the FADPred approach [1]. Table 7 shows the comparison results, indicating that the proposed method demonstrates superior performance relative to the FADPred method [1].

**Table 7:** Comparison of performance in identifying FAD binding sites in two newly discovered proteins

Classifier	True Positive	False Positive	True Negative	False Negative	Sens	Spec	Acc	MCC
Proposed Method								
Q96HE7	6	68	394	1	85.71	85.28	85.29	0.24
A3KEZ1	6	5	400	0	100	98.77	98.78	0.73
FADPred								
Q96HE7	7	282	179	0	100	38.83	39.74	0.1
A3KEZ1	6	83	321	0	100	79.46	79.46	0.23

### Identification of new FAD binding sites in electron transport protein

In this part, we applied our method for prediction of FAD binding sites in electron transport human proteins. The testing dataset retrieved from Swiss-Prot [34], which is a famous protein database. After using BLAST to remove sequence similarity more than 30 %, the rest of dataset contained 100 proteins, which including 21985 amino acids. Then our model can found 1136 FAD binding sites from dataset. Thus our research can help biologists discover some new FAD binding sites in electron transport proteins.

### Web server for predicting FAD binding sites in electron transport protein

The web server FAD-ETC.-RBF was built for presenting our method in this study. FAD-ETC.-RBF trained for the identification of FAD binding sites in electron transport proteins by using QuickRBF classification based on PSSM profiles and SAAPs. The web server can be access at http://140.138.155.226/~kahn/Bioinformatics/. We developed friendly web interface including many page menus that users can easily use to retrieve information and submit their sequences. Moreover, the users just wait for the short time to receive the prediction result because the performance of this server is especially fast. In the result page, users can easily check the results because the amino acids predicted were displayed as different colors. According to this web server, biologists can discover new FAD binding sites in electron transport protein to understand clearly the operating mechanism of electron transport chains.

## Conclusions

Predicting FAD binding sites in electron transporters is vital in helping biologists clearly understand the operating mechanisms of electron transport chains and Complex II. In this study, we developed a method based on PSSM profiles and SAAPs for identifying FAD binding residues in electron transport proteins. We used the independent data set to evaluate the performance of the proposed method, which achieved an accuracy of 69.84 %. We compared the performance of the proposed method in analyzing two newly discovered electron transport protein sequences with that of the general FADPred approach of Mishra and Raghava. We observed that the accuracy of the proposed method improved by 9 %–45 % and its MCC was 0.14–0.5. The proposed method can serve as an effective tool for predicting FAD binding sites in electron transport proteins and can help biologists understand the functions of the electron transport chain, particularly those FAD binding sites. We also developed a web server for the method described in this paper.

The contributions of this study provide a basis for further research that can enrich the field. However, this study still has some limitations related to the small sample size and limited time. The number of suitable FAD binding proteins in electron transport chains was not sufficient, potentially affecting the performance of the proposed method. To create a more effective model, we must identify additional FAD binding proteins in electron transport proteins. Doing so can enable us to conduct a comparative study and enhance prediction performance.

## Abbreviations

ATP, adenosine triphosphate; AUC, area under curve; BLOSUM, block substitution matrix; FAD, flavin adenine dinucleotide; MCC, Matthew’s correlation coefficient; NADH, nicotinamide adenine dinucleotide; PAM, percent accepted mutation; PSSM, position specific scoring matrix; RBFN, radial basis function network; ROC, receiver operating characteristic; SAAPs, significant amino acid pairs
